# Outcomes of obesity medications in those with low response to a low-energy diet meal replacement programme: An observational study

**DOI:** 10.1016/j.obpill.2026.100245

**Published:** 2026-01-03

**Authors:** James Shand, Yannan Jiang, Rinki Murphy

**Affiliations:** a Department of Medicine, Faculty of Medical and Health Sciences, The University of Auckland, Private Bag 92019, Auckland, New Zealand; b Te Mana Ki Tua, Specialist Weight Management Service, Counties Manukau Health New Zealand, Te Whatu Ora, 149 Kirkbride Rd, Auckland, New Zealand; c Maurice Wilkins Centre for Biodiscovery, Private Bag 92019, Auckland, New Zealand; d Department of Statistics, Faculty of Science, The University of Auckland, Private Bag 92019, Auckland, New Zealand

**Keywords:** Meal replacement, Obesity, Obesity medication

## Abstract

**Background:**

It is not known how individuals respond to liraglutide or naltrexone/bupropion following low-response to a structured meal replacement low energy diet (MR-LED) programme.

**Methods:**

This was a retrospective observational study conducted at a specialist weight management service using MR-LED with intensive behavioural therapy (IBT). Adults were considered for obesity medication if they did not lose at least 5 % body weight in the first 4 weeks of the intensive MR phase, 10 % after the total 12 week MR phase or regained >4 kg either during the stepped food reintroduction or weight maintenance phases. Eligible individuals were offered liraglutide 3.0 mg (LIRA) or naltrexone/bupropion (NB32) for 13 weeks (including dose titration). The primary outcome was the proportion of individuals achieving ≥5 % weight reduction after 13 weeks of treatment. Completers were defined as those who had a body weight measured between 10 and 16 weeks and did not discontinue the medication before this period.

**Results:**

Of 114 people offered obesity medications, 85 accepted (67 LIRA, 18 NB32). 46 individuals completed 13 weeks’ treatment and 11 (24 %) achieved ≥5 % weight reduction. Among completers (54 %), the mean weight change was −4.4 kg (−2.4 %), and was −2.7 kg (−1.5 %) for the total treated cohort using last weight carried forward on missing data. Those achieving ≥5 % weight reduction were more likely to be male and had lower prevalence of diabetes, binge eating, anxiety or depressive symptoms at baseline.

**Conclusion:**

People who do not respond to a MR-LED with IBT have lower than anticipated weight reduction on subsequent treatment with first-generation obesity medications.

## Graphical abstract

**Figure:**
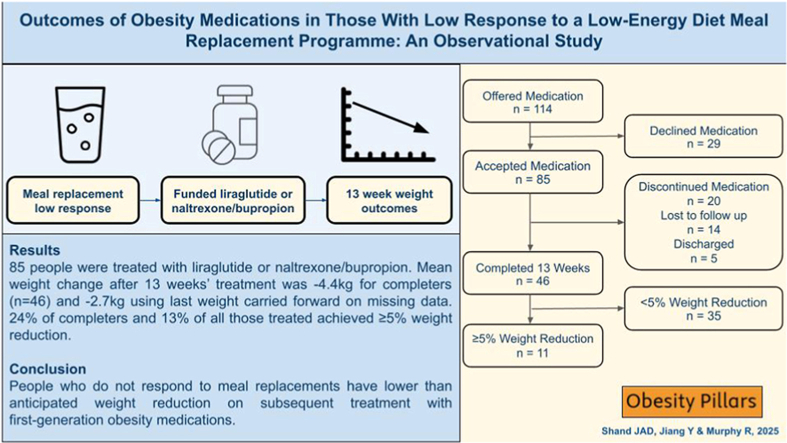

## 1 Introduction

Obesity is a chronic, relapsing condition associated with significant morbidity and mortality [1]. In New Zealand, over two thirds of adults have overweight or obesity, with disproportionately higher prevalence in Māori and Pacific people and those living in socioeconomic deprived areas [2]. Weight loss of 5–10 % is associated with clinically meaningful improvements in several parameters including glycaemic control, blood pressure and lipid profile [3]. However, achieving and maintaining even modest weight reduction is challenging due to counter-regulatory neurohormonal systems that defend weight [4]. In addition, habitual maladaptive eating behaviours such as emotional eating and binge-eating interact with an obesogenic food environment rich in ultra-processed foods and sugary beverages, making it more challenging to achieve and maintain weight loss.

Structured meal replacement low-energy diet (MR-LED) programmes are endorsed by clinical guidelines because they produce superior results compared to food-based caloric restriction [5]. Ketosis induced in the intensive phase, alongside the ease of MR-LED and food stimuli narrowing, reduces gut activation, hunger and food cravings to enable rapid and significant weight loss [6,7]. Longer term weight maintenance requires stepped reintroduction of less ultra-processed, more satiating foods alongside behavioural strategies such as food preparation and planning for regular meal times and managing triggers for impulsive eating [8]. However, a proportion of individuals fail to respond adequately to the intensive meal replacement phase or experience early weight regain during food reintroduction or weight maintenance phases. For these patients, adjunctive use of obesity medications may help overcome physiological and behavioural barriers to weight control.

Obesity medications act primarily through neurohormonal modulation and are effective adjuncts to lifestyle interventions [9]. At the time of this study, no obesity medications were publicly funded in New Zealand, and their cost was prohibitive for most individuals living with obesity. The two most potent obesity medications registered and available in New Zealand at the time of conducting this study were liraglutide 3.0 mg (LIRA), a once daily subcutaneous injectable glucagon-like peptide receptor 1 receptor agonist (GLP1RA) that reduces appetite and slows gastric emptying [10] and naltrexone 32mg/bupropion 360 mg (NB32), an oral combination tablet taken twice daily that reduces food cravings and reward-driven eating [11]. In large clinical trials, each agent has demonstrated mean weight loss of approximately 9 % [12,13]. Prioritising funded obesity medications for individuals who do not respond to intensive lifestyle interventions such as MR-LED may represent an equitable approach to ensure access to effective obesity treatment.

There is no published data on the effectiveness of obesity medications in those with low-response to MR-LED. LIRA has been studied as an adjunctive treatment in those who responded to initial MR-LED by losing at least 5 % body weight [14]. In this study, only the responders to MR-LED were randomised to placebo or liraglutide, and the liraglutide group demonstrated additional mean weight loss of 6 % by 12 weeks and 8 % by 20 weeks, maintained out to 56 weeks on treatment. One real-world study described obesity medication use after MR-LED but medications were not prescribed systematically and their use was unrelated to prior MR-LED non-response [15].

This study aims to describe the uptake and early weight outcomes of individuals offered funded LIRA or NB32 following low-response to a structured MR-LED delivered in a publicly funded specialist weight management service.

## 2 Methods

### 2.1 Study setting and design

This was a retrospective observational study conducted at Te Mana Ki Tua (TMKT), a publicly funded specialist weight management service established in 2023 in South Auckland, New Zealand, a region with high levels of socioeconomic deprivation and obesity-related comorbidity [16,17]. We describe weight outcomes in individuals offered either NB32 or LIRA by TMKT between 1 November 2023 and 31 May 2025 in the context of starting a MR-LED programme.

### 2.2 TMKT MR-LED program

TMKT is a multidisciplinary service offering fully-funded intensive weight management support. The inclusion criteria for this program were body mass index (BMI) > 35 kg/m^2^ and either the presence of an obesity complication likely to respond to 10–15 % weight loss or weight loss required to access an elective medical or surgical procedure. During its first 12 months of operation, TMKT largely invited people who had been declined public-funded bariatric surgery. A small number of additional individuals were invited who required weight loss to access corneal transplant surgery or who were young adults (aged 18–27 years) with type 2 diabetes and a community haemoglobin A1c > 75 mmol/mol (9 %).

The TMKT MR-LED structured program began with 12 weeks’ replacement of food with low energy meal replacement products, providing around 800–900 kcal/day with adjustments based on body weight and protein requirements. Individuals began the intervention in monthly cohorts of 10–15 people. This phase was followed by a further 12 weeks of stepwise food reintroduction, then 6 months of ongoing behavioural support for weight maintenance. Participants were invited to 18 group sessions and received 5 multidisciplinary team clinic contacts across 12 months. At each clinic contact, medical staff reviewed medications and treated metabolic comorbidities. The first group of participants received Optifast products with the next 11 groups receiving Counterweight Plus products before the service returned to using Optifast products. The two MR-LED interventions are similar though Optifast requires individuals to consume an additional two cups of non-starchy vegetables in addition to the MR-LED products. All MR-LED products were provided by the service at no cost to participants.

LIRA or NB32 were offered systematically by medical staff to those who did not respond to the MR-LED based on pre-specified criteria.

### 2.3 Medication criteria

TMKT-funded obesity medications (LIRA or NB32) were systematically offered based on pre-specified criteria for either early MR-LED non-response defined by: <5 % weight loss during the first 4 weeks of MR-LED; or <10 % weight loss by 12 weeks MR-LED; or weight regain >4 kg between 12 and 36 weeks of the programme.

The choice of obesity medication, between NB32 (oral twice daily) and LIRA (subcutaneous daily injection), was made collaboratively between the participant and clinician, considering contraindications, comorbidities, side effect profiles, and patient preferences regarding mode of administration. Medications were funded by the service at no cost to participants.

### 2.4 Medication titration protocol

To optimise tolerability, both LIRA and NB32 were initiated at a low dose with gradual dose increases over a period of 4 weeks. To ensure treatment is not continued in those who do not respond, and to optimise the benefit:risk ratio, guidelines suggest stopping either medication if there is less than 5 % weight loss after 12 weeks of maximum tolerated dose (excluding the titration phase) [[18], [19], [20]]. Given that the magnitude of early weight loss response to medication predicts the magnitude of weight loss response at 1 year [19], [20], assessing weight loss efficacy of at least 5 % after 13 weeks rather than after 16 weeks, should predict better responders to long-term treatment. Hence, in this study, weight loss efficacy was assessed at 13 weeks from the time of medication initiation (including the titration phase).

### 2.5 Data collection and outcome measures

Baseline data were collected at the start of the TMTK programme, including demographic information, body weight in kg, blood pressure (BP), obesity complications and questionnaire data. Obesity complications were assessed using the King’s Obesity Staging Score (KOSS) - a questionnaire regarding the prevalence and severity of 9 obesity complications. The three psychological screening tests used were: Patient Health Questionnaire 9 (PHQ9, a 9-point questionnaire investigating depressive symptoms) [21], Generalised Anxiety Disorder 7 (GAD7, a 7-point questionnaire regarding anxiety) [22] and Binge Eating Disorder Screening 7 (BEDS7, a 7- point questionnaire on the frequency of binge eating behaviours) [23].

KOSS staging and psychological questionnaires were repeated at each 3-monthly clinic contact. Weight and BP were assessed at every group session, and during individual clinic visits particularly at the start of an obesity medication and at 13 weeks after initiation.

The primary outcome was the proportion of completers achieving ≥5 % weight loss after 13 weeks on either TMKT-funded medication (LIRA or NB32). Completers were defined as those who had a body weight measured between 10 and 16 weeks (i.e. week 13 ± 3 weeks) and did not discontinue the medication during this timeframe. Secondary outcomes included the uptake of medications amongst those eligible, medication discontinuations, those lost to follow up, mean and % weight change at 13 weeks, and the characteristics of those who had weight reductions of ≥5 % vs those with weight reductions of <5 %.

### 2.6 Statistical analysis

Descriptive statistics were used to summarise baseline demographics and clinical characteristics of all study participants. Continuous variables are presented as mean (SD), and categorical variables are presented as n (%). Statistical analysis was undertaken to compare groups using the *t*-test for continuous variables and the Chi-squared or Fisher’s exact tests for categorical variables. Statistical significance level was set at 5 % (two-sided).

All analyses were conducted using R (version 4.5.0, R Core Team 2025).

### 2.7 Ethics

The study was conducted in accordance with The Code of Ethics of the World Medical Association (Declaration of Helsinki). It was approved by the Auckland Health Research Ethics Committee (AHREC AH28855) and the Counties Manukau Health research office gave localities approval (#2056).

## 3 Results

**Fig. 1:**
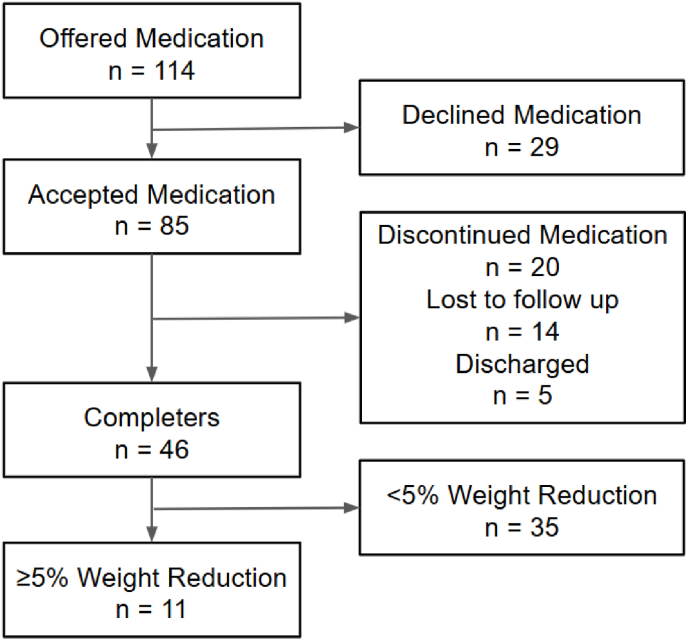
Patient flow diagram.

During the 19-month study period between 1st November 2023 to 31st May 2025, 114 individuals were offered obesity medications, of whom 29 (25 %) declined. Acceptance rates were 13/15 (87 %) for those offered at 4 weeks, 47/73 (64 %) offered at the completion of the 12 week MR-LED phase, and 25/26 (96 %) offered after 12 weeks. The study cohort included 85 participants who accepted an obesity medication, of whom 67 (79 %) received LIRA and 18 (21 %) NB32. 46 (54 %) individuals in the study cohort completed 13 weeks’ treatment and had a measured weight available between 10 and 16 weeks of treatment, of whom 11 achieved ≥5 % weight reduction (Fig. 1).

Table 1 presents the baseline characteristics of those who accepted versus declined obesity medication and Table 2 shows medication acceptance rates by key groups. Individuals who declined medication were more likely to have been offered treatment immediately after the 12-week MR-LED and were less likely to have type 2 diabetes. There were no differences in accepting or declining medications by ethnicity, gender, age or baseline psychological screening scores.

**Table 1:** Baseline characteristics of those who accepted versus declined obesity medications.

		Accepted Medication (n = 85)	Declined Medication (n = 29)	p values
**Age, years**		45.6 (10.8)	49.5 (9.8)	0.07
**Baseline Weight, kg**		151.1 (42.1)	137.5 (32.2)	0.08
**Baseline BMI, kg/m^2^**		52.1 (13.2)	47.1 (10.0)	**0.04**
**Female**		53 (62 %)	16 (55 %)	0.64
**Ethnicity**	*Māori*	22 (26 %)	6 (21 %)	0.91
	*Pacific*	49 (58 %)	19 (66 %)	
	*European*	7 (8 %)	2 (7 %)	
	*Other*	7 (8 %)	2 (7 %)	
**Phase**	*During MR-LED*	13 (15 %)	2 (7 %)	**0.003**
	*At 12 weeks*	47 (55 %)	26 (90 %)	
	*Later*	25 (29 %)	1 (3 %)	
**Psychological screening**	*PHQ9*	5.8 (5.6)	5.6 (4.8)	0.89
	*GAD7*	4.3 (4.8)	4.2 (4.6)	0.93
	*BEDS7*	2.8 (4.1)	2.6 (3.8)	0.76
**Diabetes status**	*Diabetes*	50 (59 %)	14 (48 %)	**0.04**
	*Pre-diabetes*	26 (31 %)	8 (28 %)	
	*Normoglycaemia*	9 (11 %)	7 (24 %)	
**HbA1c, mmol/mol**		54.9 (16.6)	55.0 (17.9)	0.97

Data are presented as mean (SD) or n (%). p values were calculated using t-tests for continuous variables and Chi-square tests for categorical variables. Bold p values are significant at p < 0.05.

**Table 2:** Rates of medication acceptance by key baseline factors.

		Offered medication (n)	Accepted medication (n (%))
**Gender**	*Female*	69	53 (77 %)
	*Male*	45	32 (71 %)
**Ethnicity**	*Māori*	28	22 (79 %)
	*Pacific*	68	49 (72 %)
	*European*	9	7 (78 %)
	*Other*	9	7 (78 %)
**Phase**	*During MR-LED*	15	13 (87 %)
	*At 12 weeks*	73	47 (64 %)
	*Later*	26	25 (96 %)
**Diabetes status**	*Diabetes*	64	50 (78 %)
	*Pre-diabetes*	34	26 (76 %)
	*Normoglycaemia*	16	9 (56 %)

The mean (SD) baseline weight of those who commenced medication was 151.1 (42.1) kg with a corresponding BMI of 52.1 (13.2) kg/m^2^. The cohort was 62 % female (n = 53), with a mean (SD) age of 45.6 (10.8) years. 90 % of those receiving an obesity medication had dysglycemia (59 % type 2 diabetes, 31 % pre-diabetes). The mean baseline PHQ-9 score of 5.8 indicates at least mild depressive symptoms, with 21 % (18/85) of participants having moderate depressive symptoms based on a PHQ-9 score ≥10.

20 participants (24 %) discontinued the medication within 13 weeks due to side effects: 10/18 (56 %) prescribed NB32 and 10/67 (15 %) prescribed Liraglutide. Another 19 participants (22 %) were either discharged or lost to follow-up. 46 participants reported taking the medication and had a weight measured between weeks 10 and 16 (i.e. completers).

11 people achieved the primary outcome of ≥5 % weight loss at 13 weeks (24 % of completers and 13 % of the total treated cohort). 2 people had weight reductions of >10 %, both of whom started LIRA during the intensive MR-LED phase. The mean (SD) weight change (% reduction) at 13 weeks was −4.4 (8.1) kg (−2.4 %) for completers, and −2.7 (7.1) kg (−1.5 %) for the total treated cohort using last weight carried forward on missing data (p values for both <0.001).

Table 3 shows the characteristics of those who achieved ≥5 % weight reduction compared to those who either did not reach 5 % weight loss and/or did not complete. Those with at least 5 % weight reduction after obesity medication initiation were on average of higher starting weight and were more likely to be male. They had lower prevalence of baseline type 2 diabetes, and lower scores for depression, anxiety and binge eating on PHQ9, GAD7 and BED7 respectively.

**Table 3:** Characteristics of those who commenced an obesity medication by their weight loss outcome.

		≥5 % weight reduction n = 11	<5 % weight reduction (completers) n = 35	<5 % weight reduction or non-completer n = 74	p values
**Age, years**		46.0 (8.3)	46.7 (11.2)	45.5 (11.2)	0.81^a^ 0.90^b^
**Baseline weight, kg**		187.6 (63.7)	143.7 (31.5)	145.7 (35.5)	**0.05^a^** 0.12^b^
**Baseline BMI, kg/m^2^**		59.1 (21.7)	51.1 (9.6)	50.6 (10.6)	0.28^a^ 0.25^b^
**Female**		3 (27 %)	25 (71 %)	50 (68 %)	**0.02^a^** **0.03^b^**
**Ethnicity**	*Māori*	6 (55 %)	12 (34 %)	16 (22 %)	0.47^a^ 0.1^b^
	*Pacific*	4 (36 %)	18 (51 %)	45 (61 %)	
	*European*	1 (9 %)	2 (6 %)	6 (8 %)	
	*Other*	0 (0 %)	3 (9 %)	7 (9 %)	
**Phase**	*During MR-LED*	2 (18 %)	3 (9 %)	11 (15 %)	0.44^a^ 0.37^b^
	*At 12 weeks*	4 (36 %)	19 (54 %)	43 (58 %)	
	*Later*	5 (45 %)	13 (37 %)	20 (27 %)	
**Mode of delivery**	*Group session*	9 (82 %)	34 (97 %)	72 (97 %)	0.14^a^ 0.13^b^
	*1 to 1*	2 (18 %)	1 (3 %)	2 (3 %)	
**Psychological screening**	*PHQ9*	3.1 (3.4)	5.8 (5.8)	6.2 (5.8)	0.06^a^ **0.04^b^**
	*GAD7*	2.1 (2.7)	4.5 (5.1)	4.7 (5.0)	**0.05^a^** **0.03^b^**
	*BEDS7*	0.6 (1.2)	3.1 (4.1)	3.1 (4.2)	**0.004^a^** **0.0004^b^**
**Diabetes**	*Diabetes*	2 (18 %)	23 (66 %)	48 (65 %)	**0.02^a^** **0.01^b^**
	*Pre-diabetes*	6 (55 %)	9 (26 %)	20 (27 %)	
	*Normoglycaemia*	3 (27 %)	3 (9 %)	6 (8 %)	
**HbA1c, mmol/mol**		44.8 (7.4)	56.2 (16.9)	56.4 (17.1)	**0.003^a^** **0.002^b^**

Data are presented as mean (SD) or n (%). p values were calculated using t-tests for continuous variables and Chi-square tests or Fisher’s exact tests for categorical variables. Superscript a (a) refers to significance testing between those with ≥5 % weight reduction and completers with <5 % weight reduction. Superscript b (b) refers to significance testing between those with ≥5 % weight reduction and those who either achieved <5 % weight reduction or were non-completers. Bold p values are significant at p < 0.05.

In the study cohort, 43/67 people who were prescribed LIRA completed the treatment with 11 achieving ≥5 % weight reduction. 3/18 people who were prescribed NB32 completed the initial treatment period, with none achieving ≥5 % weight reduction.

## 4 Discussion

To our knowledge, this is the first study to evaluate obesity medication treatment response in those with low response to MR-LED. This study shows only 75 % uptake and low effectiveness of first-generation obesity medications NB32 and LIRA in a multi-ethnic cohort of people with mean BMI of 52.1 kg/m^2^, who did not respond to a structured, group-based MR-LED with multidisciplinary team support.

The acceptance of funded obesity medication was lower than anticipated. Cost is frequently cited as a significant barrier to the uptake of these treatments amongst people living with obesity [24]. However, despite the provision of obesity medications free of charge under specialist MDT care, 25 % of those offered declined. The baseline characteristics of those accepting vs declining obesity medication were similar, with no significant differences in age, gender, weight, ethnicity or baseline psychological distress. Those with type 2 diabetes were more likely to accept an obesity medication which may be due to a higher baseline pill burden and increased familiarity with taking medications. The majority of those who declined medication were offered treatment immediately after completing the 12-week MR-LED intervention, with very high acceptance rates of medication offered at other times. It is possible that a limited response to a first-line intervention led to reduced trust in subsequent offered treatments.

Of those who started an obesity medication, 46 % did not complete 13 weeks’ treatment, with higher rates of intolerable side effects and medication discontinuation than were seen in the much longer trials of LIRA and NB32 [11,12]. The reasons for this difference are unclear but may relate to the high-risk nature of the population treated or disengagement following a lack of early obesity medication response in the context of prior MR-LED non-response. Additionally, the TMKT medication use protocol did not require frequent prescriber follow-up during the dose titration period which may have also led to discontinuations. 87 % of those who started an obesity medication either did not complete 13 weeks’ treatment or did not achieve 5 % weight loss. Compared to those achieving 5 % weight loss, this group had higher levels of psychological distress, binge eating behaviour and type 2 diabetes. It is likely that these factors influenced the response to the prescribed obesity medication.

Low-response to MR-LED may reflect adherence challenges and/or medication hesitancy. All patients were offered multiple contacts with the multidisciplinary staff as part of the group-based program, for medication related side effects to be reported. However, it is possible that some discontinuations due to side effects could have been avoided with more frequent proactive medication-related contact and intervention. Future obesity medication initiation could include witnessed first injection in clinic to overcome injection-related hesitancy to LIRA. Those individuals might respond to more potent GLP1RA such as semaglutide or tirzepatide that are administered once weekly or to bariatric surgery.

The 13-week duration of exposure to each medication in this study is shorter than the recommended 12 weeks on maximum tolerated dose before assessing the minimum 5 % response threshold which could have reduced the observed response rate. However, pooled data from clinical trials shows 12 week (including the dose titration period) 5 % weight loss rates of 58 % for NB32 and 59 % for LIRA [19.20]. In comparison, only 13 % of our cohort and 24 % of completers achieved 5 % weight loss despite an extra week of exposure to these obesity medications.

Several factors may explain the low additional mean weight loss observed with LIRA or NB32 among low-responders to MR-LED. Firstly, low-response to a structured MR-LED intervention may indicate underlying physiological, psychological, behavioural or cultural factors making weight loss more challenging. Low-responders to structured MR-LED programs have not been systematically studied to assess whether they respond similarly to more potent obesity medications such as semaglutide, tirzepatide, as those who do respond to MR-LED, at least in the short term. Secondly, 59 % of our cohort had type 2 diabetes which can attenuate the response to obesity treatment [25]. Thirdly, our cohort had high rates of psychological distress which is often an exclusion criterion for obesity medication trials and the impact of this has not been well-studied. In the only other published study of LIRA after MR-LED, 1.9 % of participants had a PHQ9 ≥10, as compared to 21 % of our cohort [14]. It is possible that higher anxiety, depression and binge-eating behaviour may attenuate the response to first generation obesity medications. Fourthly, our population had a mean baseline body weight of 151.1 kg, as compared to 106.2 kg and 99.5 kg in the respective landmark LIRA and NB32 trials [11,12]. However, contrary to expectation, those who did achieve ≥5 % body weight after medications in our study had higher baseline body weight than those who did not. Finally, South Auckland is a diverse and socio-economically deprived region. There are no studies evaluating the impact of deprivation on obesity response, yet poverty in itself is known to contribute to obesity through food insecurity, lack of access to healthy foods and barriers to physical activity [26].

## 5 Limitations

This research has a number of limitations that should be considered. Firstly, the study was observational in nature which means bias is possible. Secondly, the sample size was small, with only 85 people treated. Thirdly, follow up time was short and it is possible that more people would have responded following a greater duration of treatment. Fourthly, there was a high dropout rate despite intensive support being offered by a multidisciplinary team. Finally, the cohort treated were living with very high body weights and levels of physical and psychological comorbidity. These findings may not be generalisable to other populations.

## 6 Conclusion

There is low weight loss response to first generation obesity medications LIRA and NB32 when offered to people living with very high body weights and complex comorbidities who have not responded to a structured MR-LED program. Only 13 % achieved 5 % additional weight loss after 13 weeks treatment with these medications. Further studies are required to understand the mechanisms of non-response and whether such individuals benefit from newer more potent medications such as semaglutide or tirzepatide, and/or bariatric surgery.

## 7 Takeaways

● Individuals who do not respond to an initial MR-LED do not achieve significant additional weight reductions following 13 weeks of liraglutide or bupropion/naltrexone
● Real-world experience shows low (75 %) uptake of liraglutide or bupropion/naltrexone with high rates of discontinuation (54 %) in the first 13 weeks, when provided free of charge in a specialist weight management service
● Further research is needed on whether MR-LED non-responders achieve additional weight loss with more potent, newer generation obesity medications such as semaglutide or tirzepatide

## Author contribution

JS, YJ and RM were responsible for conceptualization and methodology. Data collection was performed by JS and RM. Statistical analysis was by JS and YJ. JS wrote the first draft. JS, YJ and RM all reviewed, edited, and approved the final submission and publication.

## Ethics

The study was conducted in accordance with The Code of Ethics of the World Medical Association (Declaration of Helsinki). It was approved by the Auckland Health Research Ethics Committee (AHREC AH28855) and the Counties Manukau Health research office gave localities approval (#2056).

## Declaration of artificial intelligence (AI) and AI-assisted technologies

During the preparation of this work the authors did not use AI.

## Source of funding

This research was supported by a grant from the 10.13039/501100018883Maurice Wilkins Centre.

## Declaration of competing interest

JS has received speaking honoraria from Novo Nordisk.

YJ has nothing to disclose.

RM has received speaking and advisory board honoraria from Eli Lilly, Novo Nordisk, Boehringer Ingelheim.
